# Dupilumab for the treatment of atopic dermatitis and alopecia areata with incidental improvement of trachyonychia

**DOI:** 10.1016/j.jdcr.2025.03.040

**Published:** 2025-06-14

**Authors:** Dana H. Chung, Grace A. Osborne, Julie E. Mervak, Jennifer B. Mancuso

**Affiliations:** a University of Michigan Medical School, Ann Arbor, Michigan; b Department of Dermatology, University of Michigan, Ann Arbor, Michigan; c Department of Pediatrics, University of Michigan, Ann Arbor, Michigan

**Keywords:** alopecia areata, atopic dermatitis, dupilumab, trachyonychia

## Introduction

Trachyonychia is an inflammatory disease of the nail matrix characterized by longitudinal ridging or diffuse fine pitting of the nail plate. The rough appearance, seen in the opaque variant of trachyonychia, is commonly referred to as “sandpaper nails.” In milder cases, described as the shiny variant of trachyonychia, nails retain luster but present with superficial longitudinal ridging and pitting. Trachyonychia is more common in pediatric than in adult patients and is often idiopathic, but can be observed in inflammatory skin and nail conditions including psoriasis, lichen planus, atopic dermatitis, and alopecia areata (AA).^1^ We present a pediatric patient with a history of atopic dermatitis and AA whose trachyonychia significantly improved with the use of dupilumab.

## Case report

**Fig 1:**
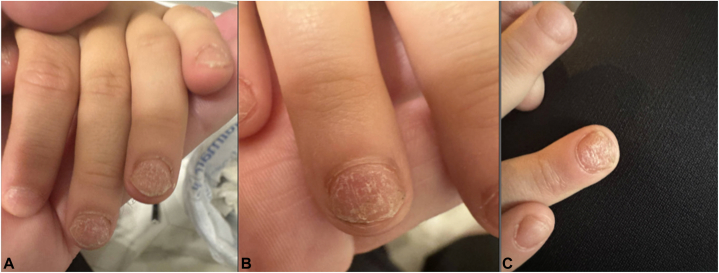
**A-C,** Irregular diffuse pitting and superficial longitudinal ridging of the fingernails with thickened hyperkeratotic cuticles consistent with trachyoychia.

A 5-year-old boy with a history of atopic dermatitis and AA, alopecia universalis subtype, presented to pediatric dermatology clinic with trachyonychia. Over a 2-year treatment period, his AA remained active despite treatment with topical steroids, topical minoxidil, fexofenadine,^2^ and a 12-week pulsed dexamethasone course. His atopic dermatitis was mild to moderate despite the appropriate use of topical steroids (triamcinolone 0.1% ointment and mometasone 0.1% ointment). Diffuse pitting of his fingernails started 1 year prior to presentation with progressive worsening leading to longitudinal ridging, brittleness, and intermittent pain. On physical examination, all 10 fingernails and his bilateral first toenails displayed superficial longitudinal ridging, fine pitting, and hyperkeratosis of the fingernail cuticles (Fig 1). The patient was started on dupilumab 300 mg subcutaneously every 4 weeks for atopic dermatitis. Due to the patient’s concomitant AA, the threshold for prescription of dupilumab was lowered from the indicated moderate to severe atopic dermatitis. This therapy was also used with the goal of treating his AA. While trachyonychia is not pathognomonic for a specific disease given that his atopic dermatitis was controlled and AA was progressive, we concluded that his nail changes were associated with AA.

**Fig 2:**
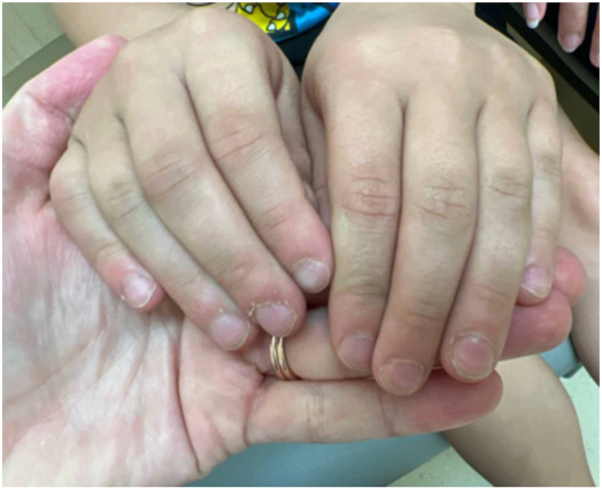
Significant improvement of pitting and superficial longitudinal ridging of the fingernails; minimal residual thickened hyperkeratotic cuticles after 3 months of dupilumab 300 mg every 4 weeks.

At 3-month follow-up, his atopic dermatitis had significantly improved and topical steroid treatment was no longer required. AA was overall stable on his scalp with some regrowth of the eyebrows and eyelashes. Notably, his nail changes had significant improvement with resolution of the superficial longitudinal ridging of the nail plates and cuticle thickening (Fig 2). No adverse effects of dupilumab were noted.

## Discussion

Trachyonychia is most often a clinical diagnosis. Histopathology is not routinely performed; however, most commonly, biopsy demonstrates focal spongiotic inflammation in the nail matrix.^3^ Based on these observations, some authors have postulated that trachyonychia may represent a focal eczematous change of the nail unit.^1^^,^^3^^,^^4^ A recent literature review noted that of 16 AA patients with trachyonychia who underwent biopsy, 14 were found to have spongiosis on histology.^1^ T helper 2 (Th2) cells produce cytokines such as interleukin (IL) 4, IL-5, and IL-13 that are associated with allergic response and atopic conditions. The Th2 pathway has been implicated as the main driver of inflammation in the pathogenesis of atopic dermatitis. Recent studies have shown that Th2 cells and related cytokines are also implicated in AA, correlating with disease severity.^5^ A frequent coexistence of AA and atopic dermatitis suggests a common pathogenesis of these conditions with the potential role of Th2 cytokines.^5^ However, the role of Th2 cells in AA is not fully understood.

Dupilumab is a fully human monoclonal antibody against the IL-4 alpha chain receptor, mitigating Th2 by blocking signaling of IL-4 and IL-13.^6^ Dupilumab is approved by the United States Food and Drug Administration to treat moderate to severe atopic dermatitis in patients aged 6 months and more.^6^ Conflicting evidence suggests that at times dupilumab can be an effective treatment for other Th2-mediated conditions, including AA, but may also worsen existing AA or trigger new onset AA.^7^

Nail changes are common in patients with AA and present mostly with pitting and trachyonychia, seen in 30% and 18% of AA patients, respectively.^8^ The presence of nail changes negatively impacts patients’ quality of life and is also correlated with disease severity.^8^ Treatment of trachyonychia is challenging as topical agents may have minimal efficacy and options such as serial intralesional triamcinolone injections of the nail matrix are invasive and painful.

In our case, treatment with dupilumab resulted in improvement of trachyonychia symptoms and appearance after 3 months. The patient also had mild regrowth of eyebrows and eyelashes. Given persistent scalp involvement of AA, we favor nail changes to be secondary to AA and improvement to be due to the initiation of dupilumab rather than spontaneous improvement. Given that the pattern of spongiosis on histopathology is driven by Th2 cytokines and that AA patients have elevation of these cytokines, we hypothesize that inhibition of IL-4 and IL-13 had direct effect on the inflammation of the nail matrix leading to improvement of AA-associated trachyonychia. We suggest that dupilumab could be considered as a treatment option in these challenging cases.

## Conflicts of interest

None disclosed.
